# Delivery of rivaroxaban and chitosan rapamycin microparticle with dual antithrombosis and antiproliferation functions inhibits venous neointimal hyperplasia

**DOI:** 10.1080/10717544.2022.2092240

**Published:** 2022-06-28

**Authors:** Peng Sun, Haoliang Wu, Hao He, Liwei Zhang, Yuanfeng Liu, Cong Zhang, Chunyang Lou, Jingan Li, Hualong Bai

**Affiliations:** a Department of Vascular and Endovascular Surgery, First Affiliated Hospital of Zhengzhou University, Henan, China; b Key Vascular Physiology and Applied Research Laboratory of Zhengzhou City, Zhengzhou, Henan Province, China; c School of Material Science and Engineering & Henan Key Laboratory of Advanced Magnesium Alloy & Key Laboratory of materials processing and mold technology (Ministry of Education), Zhengzhou, Henan Province, China; d Department of Vascular Surgery, The Second Xiangya Hospital of Central South University, Changsha, China

**Keywords:** Neointimal hyperplasia, rivaroxaban, rapamycin, chitosan microparticle, polyglycolic acid

## Abstract

Neointimal hyperplasia is a complex process after vascular interventions, acute platelet deposition and smooth muscle cell proliferation both contributed to this process. There are still no perfect solutions to solve this problem. Rivaroxaban is a novel anticoagulant that has been widely used in clinic, it has a good pharmacological effects both in vivo and in vitro. Chitosan microparticle rapamycin (MP-rapa) was fabricated, interspaces of polyglycolic acid (PGA) scaffold were used as a reservoir of MP-rapa, and the scaffold was coated with hyaluronic acid rivaroxaban (MP-rapa-riva). Scanning electronic microscopy (SEM) photographs were taken and water contact angles were measured, rat inferior vena cava (IVC) patch venoplasty model was used; patches were harvested at day 14 and examined by immunohistochemistry and immunofluorescence. SEM photographs showed the microparticles rapamycin were inside the interspace of the scaffold, hyaluronic acid rivaroxaban was also successfully coated onto the surface of the scaffold. There was a thinner neointima, fewer proliferating cell nuclear antigen (PCNA) positive cells, fewer macrophages in the MP-rapa and MP-rapa-riva grafts compared to the control PGA graft. The result showed that this scaffold with dual anticoagulation and antiproliferation functions can effectively inhibit venous neointimal hyperplasia, although this is an animal experiment, it showed promising potential clinical application in the future.

## Introduction

Neointimal hyperplasia is still the leading cause of graft failure after vascular interventions (Goel et al., 2012), it is a complex process including acute platelet deposition, inflammatory cell accumulation, and smooth muscle cell proliferation (Hristov and Weber, 2008; Lee and Roy-Chaudhury, 2009; Muto et al., 2007). Heparin, rapamycin or paclitaxel coated stents or balloons have been widely used in clinic; although these instruments contributed to a better patency rate, but none of these grafts showed a long term success in clinic (Kandzari et al., 2017; Cochrane Vascular Group, 2016; Tepe et al., 2017). Current protocols are mainly focusing on one step in the process of neointimal hyperplasia, like anti-thrombosis or anti-proliferation, this maybe the cause of the unsatisfactory result of the drug coated balloon and stent (Mehta et al., 2011). So, new methods are needed to decrease neointimal hyperplasia, especially in basic research. We recently showed a three-layered hydrogel patch with hierarchical releasing of PLGA nanoparticle drugs decrease neointimal hyperplasia in a rat inferior vena cava (IVC) venoplasty model, this hierarchical releasing system can significantly and effectively inhibit venous neointimal hyperplasia (Wei et al., 2022). But that drug delivery system is complex, so a simpler drug delivery system is needed.

Different grafts have been used in vascular surgeries, like the autologous vein, ePTFE and polyester, and pericardial patch (Bai et al., 2020). We previously showed therapeutic drugs can be conjugated to the pericardial patch (Bai et al., 2017), decellularized human saphenous vein patch (Bai et al., 2020). Polyglycolic acid (PGA) is widely used in vascular research, PGA felt is composed of PGA fibers and interspaces between them (Quint, 2020, Li et al., 2020), these interspaces can be a reservoir for drug delivery (Liu et al., 2016). Chitosan particles are easy to make and are commonly used to delivery drugs (Liang et al., 2021, Hojnik Podrepsek et al., 2020), the chitosan particles can be stored into the interspaces of PGA fibers and slowly released. Rivaroxaban is a direct FXa (Xa factor) factor inhibitor and now is widely used in the treatment of venous thromboembolism disease (Kaplovitch et al., 2020; Kearon et al., 2016; Perzborn et al., 2005; Roehrig et al., 2005), rivaroxaban can also protect the oxysterol-induced damage and inflammatory activation of the vascular endothelium (Gorzelak-Pabis et al., 2021); but there is still no research on whether rivaroxaban can be coated to the vascular graft. A previous research showed rivaroxaban plays a role in mediating tissue remodeling and inflammation (Krupiczojc et al., 2008). Rivaroxaban treatment (15 mg/kg/d) can significantly reduce the maximal aortic diameter and suppress experimental abdominal aortic aneurysm progression in ApoE-deficient (ApoE^-/-^) mice (Ding et al., 2021). It can also attenuated calcium chloride-induced abdominal aortic aneurysms in mice by inhibiting abdominal aortic remodeling and reducing peripheral inflammation (Ding et al., 2021). In a rabbit model, rivaroxaban inhibits intimal hyperplasia and smooth muscle cell proliferation at the carotid anastomosis site (Akkaya et al., 2017).

Based on these previous researches, we hypothesized that chitosan microparticle rapamycin can be loaded into the interspaces of PGA scaffold, and rivaroxaban can be coated onto the surface of the PGA scaffold to decrease venous neointimal hyperplasia in a rat inferior vena cava (IVC) venoplasty model.

## Materials and methods

### Scanning electronic microscopy (SEM) photograph and water contact angles (WCA) measurement

PGA scaffold, PGA scaffold filled with collagen-1, PGA scaffold filled with chitosan microparticle rapamycin and collagen-1 (MP-rapa), PGA scaffold filled with chitosan microparticle rapamycin and collagen-1 and coated with hyaluronic rivaroxaban (MP-rapa-riva) were observed their morphology under scanning electronic microscopy (SEM), and the water contact angles (WCA) were also measured as previously described (Wang et al., 2019).

### PGA scaffold modification

The absorbable polyglycolic acid felt (0.5 mm) was bought from GUNZE medical, Japan; the felt was cut in to 1X1cm^2^ for implantation or modification.

Chitosan (F1905050, Aladdin, China) was dissolved at 0.5 wt% in 1.5% acetic acid (H1808008, Aladdin, China), once dissolved adjust pH to 4.7. 0.5% tripolyphosphate (TPP, H1808008, Aladdin, China) crosslinker solution (1 mL, with 0.5 mg rapamycin) was added into chitosan solution (3 mL) at room temperature with constant stirring as previously described. Microparticles was then stabilized by a 30 min incubation at room temperature and collected by centrifugation at 13,000 × g at 10 °C for 30 min. Collagen-1 (C8061, Solarbio, China) solution was prepared at the concentration of 0.1%. The coating of hyaluronic acid rivaroxaban was carried out as we previously described (Bai et al., 2020). The samples were immersed in a hyaluronic acid (HA) solution for 15 minutes, then washed with phosphate-buffered saline. The HA coated samples were then immersed in a rivaroxaban solution (1 mL, 2 mg/mL) that had also been advance-activated in water-soluble carbodiimide solution for 15 minutes, then incubated for 6 hours. Rivaroxaban was given at a dose determined by previous studies (Gigi et al., 2012; Seki et al., 2017; Álvarez et al., 2018).

### Animal model

The First Affiliated Hospital of Zhengzhou University authorized all research, and the National Institutes of Health guidelines for the care and use of laboratory animals (NIH Publication #85–23 Rev. 1985) were followed. For patch implantation, male Sprague Dawley (SD) rats (6–8 weeks old) were used as previously described (Bai et al., 2017). The inferior renal inferior vena cava (IVC) was exposed through a midline incision; on the anterior wall of the IVC, a 3 mm longitudinal venotomy was made approximately 2 mm below the level of the renal vein.

Microsurgical procedures were performed aseptically using a dissecting microscope (Nikon, Japan). Control patch (PGA scaffold filled with collagen-1), MP-rapa patch (PGA scaffold filled with chitosan nanoparticle rapamycin and collagen-1), MP-rapa-riva patch (PGA scaffold filled with chitosan nanoparticle rapamycin and collagen-1 and coated with hyaluronic rivaroxaban) were trimmed to 3 mm × 1.5 mm × 0.5 mm and implanted to the rat IVC using running 11–0 nylon sutures. The abdomen was closed with 5–0 polyester sutures after patch implantation. The patches were harvested for analysis at day 14 after patch implantation, since the neointimal thickness was consistent after day 7 in this model (Bai et al., 2017; Bai et al., 2017; Bai et al., 2016). No immunosuppressive, antibacterial, or antiplatelet medications were administered at any time. Patches were also implanted subcutaneously in rat.

### Hematoxylin and eosin (H&E) staining

Rats were anesthetized with 10% chloral hydrate (intraperitoneal injection), and tissues were fixed with transcardial perfusion of phosphate buffered saline (PBS) followed by 10% formalin. Patch was removed and fixed in 10% formalin for 12 hours. Tissues were then paraffined and sectioned (4-μm thickness). Tissue slices were deparaffinized and stained with H&E staining kit (Baso, Zhuhai, China). The neointimal thickness was the mean of measurements in three different areas.

### Immunostaining

Tissue sections were deparaffinized and were heated in a citric acid buffer (pH 6.0, Beyotime, Shanghai, China) at 100 °C for 10 min for antigen retrieval, and then incubated with primary antibodies overnight at 4 °C. After 1 hour of incubation at room temperature with secondary antibodies, the sections were stained with DAPI (Solarbio, Beijing, China) or hematoxyline to identify cellular nuclei.

### Antibodies

Primary antibodies included: anti-CD31 (R&D, AF3628; IHC, 1:100); anti-CD68 (Abcam, ab31360; IF, 1:50); anti-α-actin (Abcam, ab5694; IF, 1:200); anti-IL10 (Abcam, ab9969; IF, 1:100); antiiNOS (Abcam, ab15323; IF, 1:100); anti-TGM2 (Abcam, ab421; IF, 1:100); anti-TNF-α (Abcam, ab6671; IF, 1:100); anti-CD34 (Abcam, ab81289; IF, 1:50); anti-phospho-mTOR (ABclonal, AP0094; IF, 1:50); anti-nestin (Abcam, ab 11306; IF, 1:50). Secondary antibodies used for IF were from ABclonal, Wuhan, China.

### Statistics

The mean SEM is used to express the data. ANOVA and t-tests were used to establish statistical significance for these analyses (Prism 6; GraphPad Software, La Jolla, CA). P-values of less than 0.05 were considered significant.

## Results

**Figure 1.**
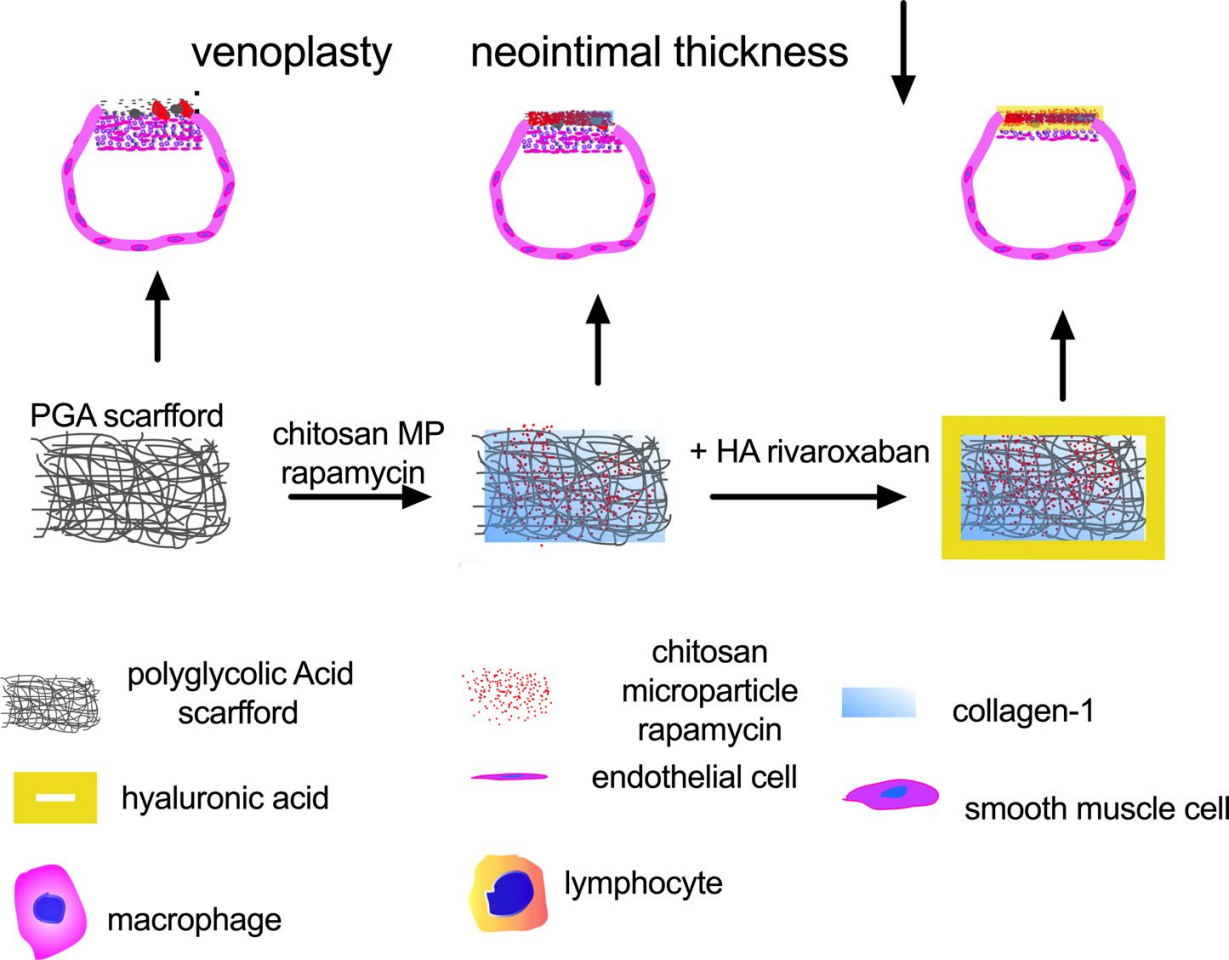
Illustration photograph showing the study design, the red color showing chitosan microparticle rapamycin. This illustration photograph was created by Dr. Hualong Bai.

**Figure 2.**
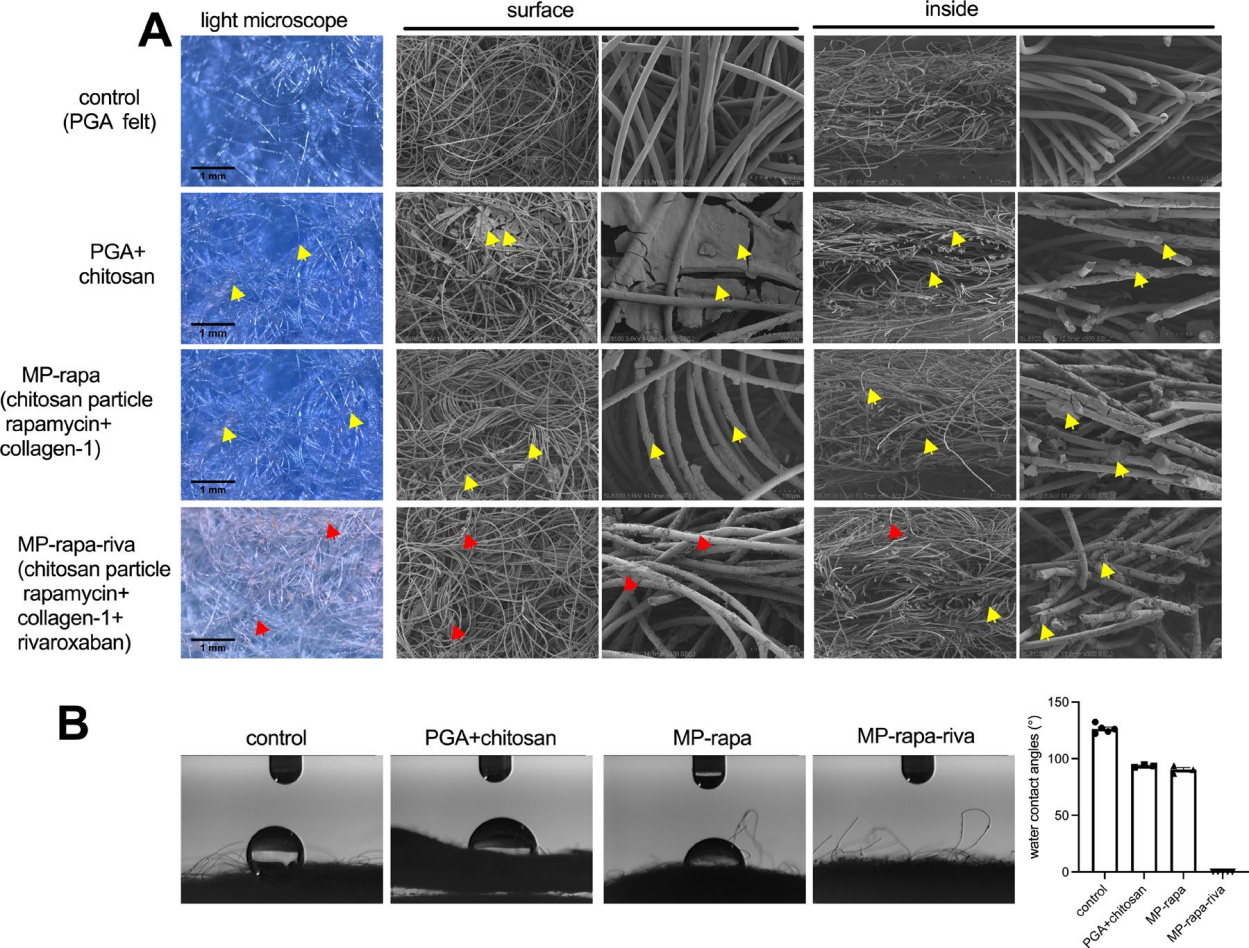
Scanning electronic microscopy (SEM) and water contact angle (WCA) of the PGA, MP-rapa (PGA + chitosan particle rapamycin + collagen-1) and MP-rapa-riva (chitosan particle rapamycin + collagen-1 + rivaroxaban). (A) SEM photograph showing the structure of the PGA fibers on the surface or inside, yellow arrow showing chitosan microparticles, red arrow showing rivaroxaban, *n* = 3-5. (B) Water contact angle of the control, MP-rapa and MP-rapa-riva groups, *n* = 3-5.

The rivaroxaban coated PGA scaffold soaked with chitosan microparticle was made as showed in the illustration photograph (Figure 1). The PGA scaffold was soaked in the mixture of collagen-1 solution and chitosan microparticle rapamycin for 5 minutes, the mixture can be recognized clearly under microscope (Figure 2A), hyaluronic acid rivaroxaban can be successfully coated to the PGA scaffold (Figure 2A). Scanning electron microscope (SEM) photographs showed chitosan microparticle rapamycin and rivaroxaban both on the surface and inside the PGA scaffold (Figure 2A). Water contact angle (WCA) showed a large angle in the control group, PGA with chitosan microparticle (PGA + chitosan) group and PGA with chitosan microparticle rapamycin (PGA + rapa) group, but after coated with hyaluronic acid rivaroxaban (MP-rapa-riva), there was a much smaller water contact angle (Figure 2B).

**Figure 3.**
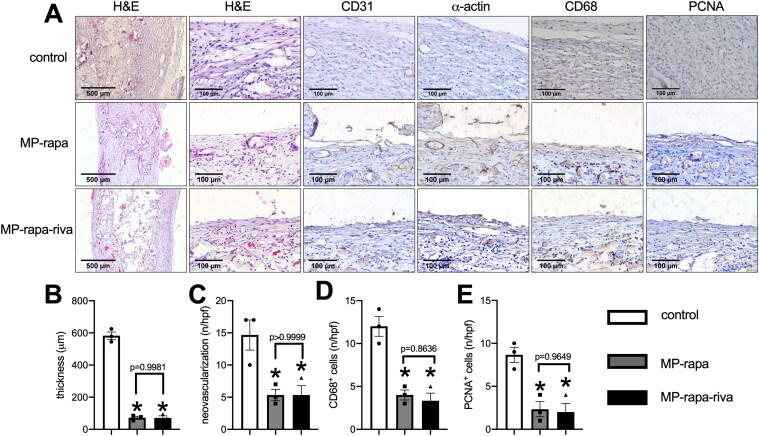
Deceased foreign body reaction and proliferation of MP-rapa and MP-rapa-riva grafts compared to the control graft in the subcutaneous implantation. (A) Photographs of hematoxylin and eosin (H&E), high power photographs of immunohistochemistry stained for CD31, α-actin, PCNA and CD68; scale bar, 500 μm or 100 μm; *n* = 3. (B) Bar graph showing the thickness of the foreign body reaction tissue; *p* < 0.0001, one-way ANOVA; *, *p* < 0.0001, Tukey’s multiple comparisons test; *n* = 3. (C) Bar graph showing the neovascularization in the new formed tissue; *p* = 0.0111, one-way ANOVA; *, *p* = 0.0175; *n* = 3. (D) Bar graph showing the CD68 positive cells in the new formed tissue; *p* = 0.0009, one-way ANOVA; *, *p* = 0.0019 and 0.0012; *n* = 3. (E) Bar graph showing the PCNA positive cells in the new formed tissue; *p* = 0.0036, one-way ANOVA; *, *p* = 0.0068 and 0.0053; *n* = 3.

These scaffolds were then implanted subcutaneously in rats and harvested at day 14, in the control group, there was a thick new tissue capsuled the graft, while there was a much thinner tissue capsuled the MP-rapa and MP-rapa-riva grafts (Figure 3A), chitosan microparticle rapamycin remained in the interspaces of PGA scaffold in the MP-rapa and MP-rapa-riva grafts (Figure 3A). There was a smaller amount of new formed capillaries (Figure 3A, 3C), smaller amount of CD68 positive cells (Figure 3A, 3D) and PCNA positive cells (Figure 3A, 3E) in the MP-rapa and MP-rapa-riva grafts; but there was no different between MP-rapa and MP-rapa-riva grafts (Figure 3A, 3E).

**Figure 4.**
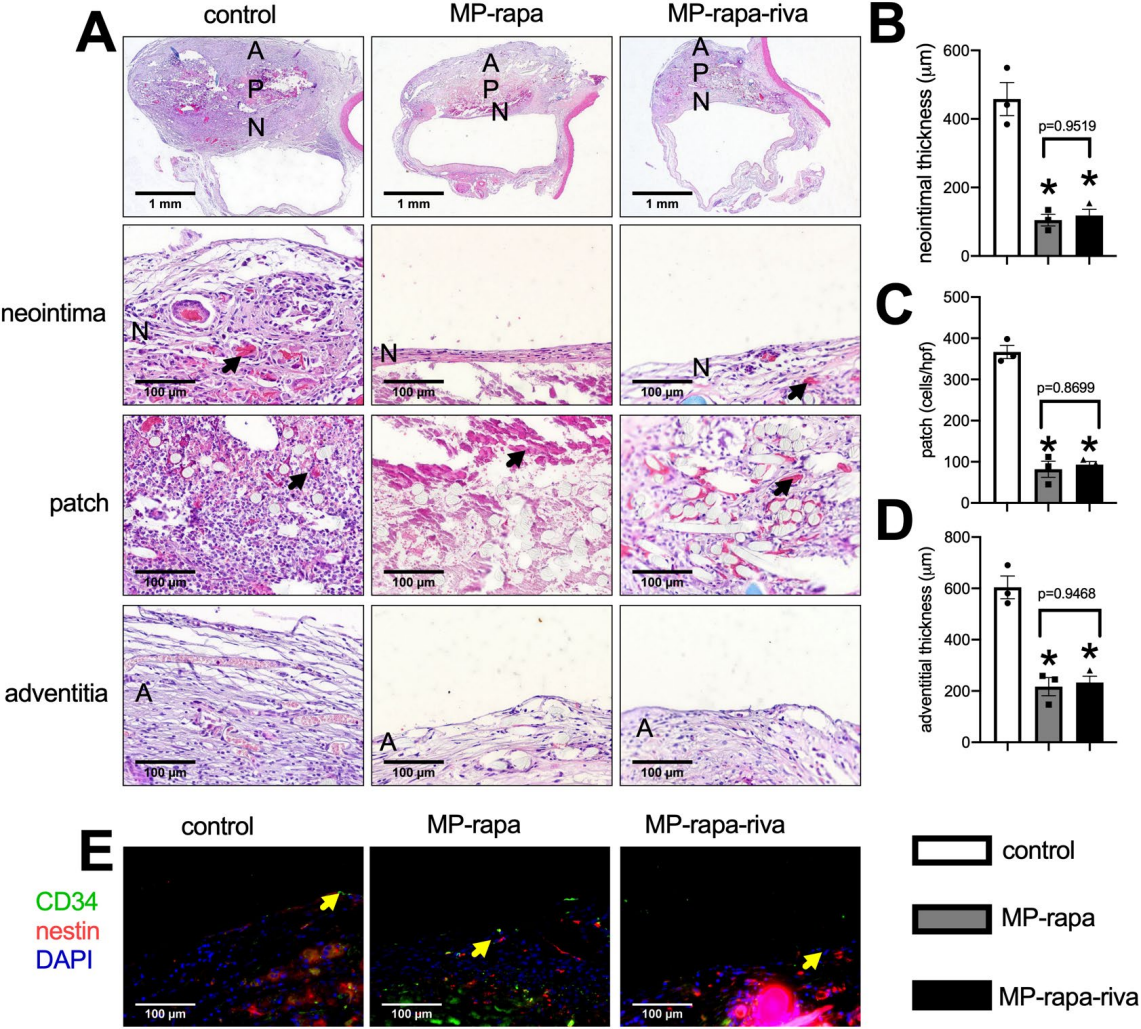
Deceased neointimal thickness in MP-rapa and MP-rapa-riva grafts compared to control graft in the rat inferior vena cava (IVC) patch venoplasty model at day 14. (A) Low power and high-power photographs of H&E staining of the patches harvested at day 14, *n* = 6. P, patch; L, IVC lumen; A, adventitial (peritoneal) surface; N, neointima. Black arrow showing the collagen and chitosan particles in the patch and neointima; scale bar, 1 mm or 100 μm; *n* = 3. (B) Bar graph showing the neointimal thickness; *p* = 0.003, ANOVA; *, *p* = 0.0005 and 0.006, Tukey’s multiple comparisons test; *n* = 3. (C) Bar graph showing the cells infiltrated into the patch (mean number of cells counted in 4 high power fields); *p* < 0.0001, ANOVA; *, *p* < 0.0001, Tukey’s multiple comparisons test; *n* = 3. (D) Bar graph showing decrease adventitial thickness; *p* = 0.0004, ANOVA; *, *p* = 0.0006 and 0.008, Tukey’s multiple comparisons test; *n* = 3. (E) Merged immunofluorescence photograph of CD34 (green), nestin (red) and DAPI (blue), yellow arrow showing the positive cell; scale bar, 100 μm; *n* = 3.

We then implanted these patches to the rat IVC, all the animals survived and all IVCs were patent with no stenosis. Patches were harvested at day 14, hematoxylin and eosin (H&E) staining showed a thick new formed adventitia and neointima in the control grafts; there was a significantly thinner neointima in both of the MP-rapa and MP-rapa-riva grafts compared to the control grafts (Figure 4A, 4B). The chitosan microparticle rapamycin can still be found in the interspaces in the MP-rapa and MP-rapa-riva grafts, there was also fewer cells infiltrated into the interspaces in the MP-rapa and MP-rapa-riva grafts (Figure 4A, 4C). There was also a significantly thinner adventitia in the MP-rapa and MP-rapa-riva grafts (Figure 4A, 4D). Immunofluorescence showed CD34 and nestin dual positive progenitor cell in the neointima in these three grafts (Figure 4E).

**Figure 5.**
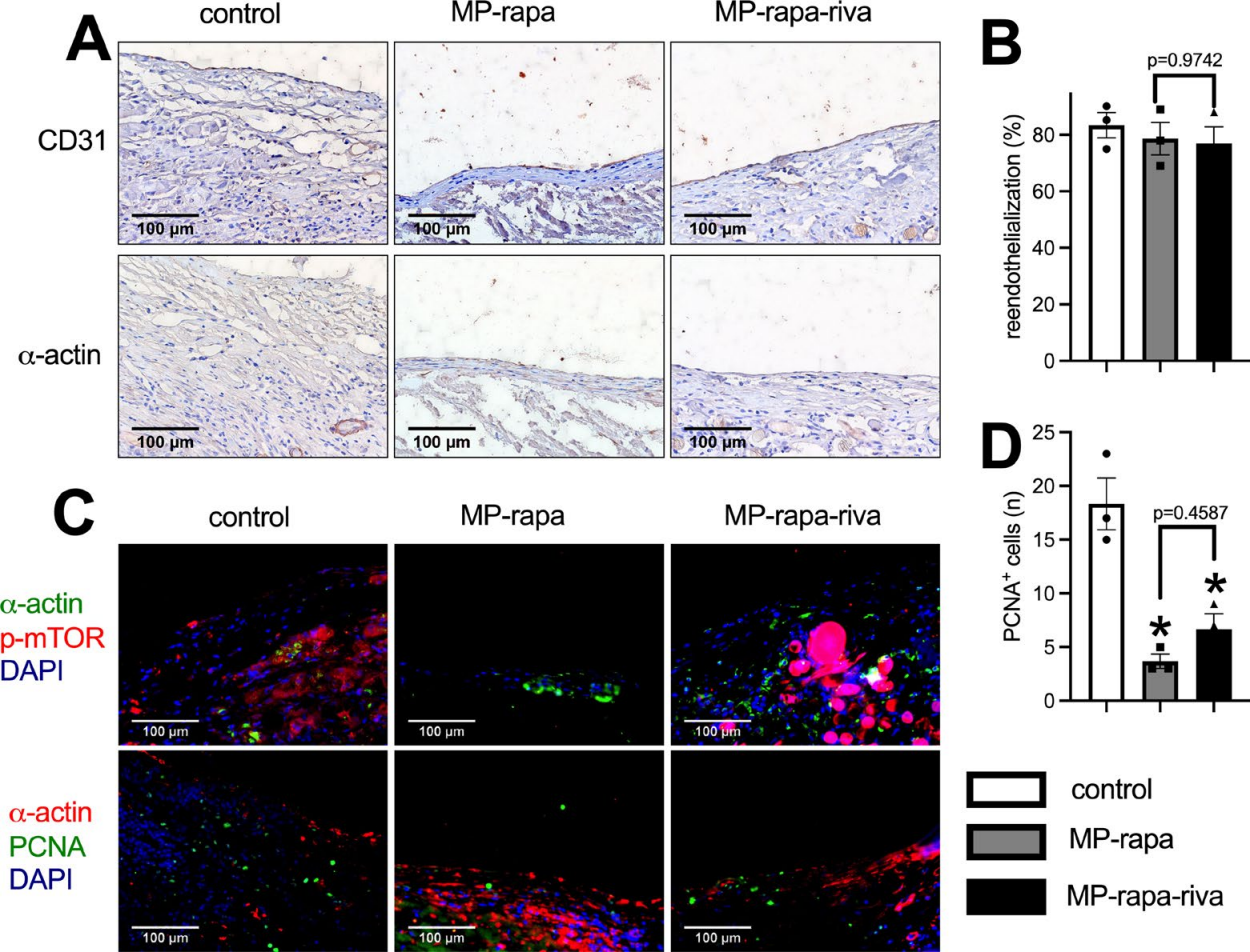
Deceased proliferation in the MP-rapa and MP-rapa-riva grafts compared to control graft in the rat IVC venoplasty at day 14. (A) Photographs of the immunohistochemistry stained for CD31 and α-actin in the control, MP-rapa and MP-rapa-riva grafts; scale bar, 100 μm; *n* = 3. (B) Bar graph showing the reendothelialization; *p* = 0.6980, one-way ANOVA; *n* = 3. (C) Photographs of the immunofluorescence of the neointima, first row, α-actin (green), p-mTOR (red) and DAPI (blue); second row, α-actin (red), PCNA (green) and DAPI (blue); scale bar, 100 μm; *n* = 3. (D) Bar graph showing the PCNA positive cells; *p* = 0.0018, ANOVA; *, *p* = 0.0019 and 0.0062, Tukey’s multiple comparisons test; *n* = 3.

**Figure 6.**
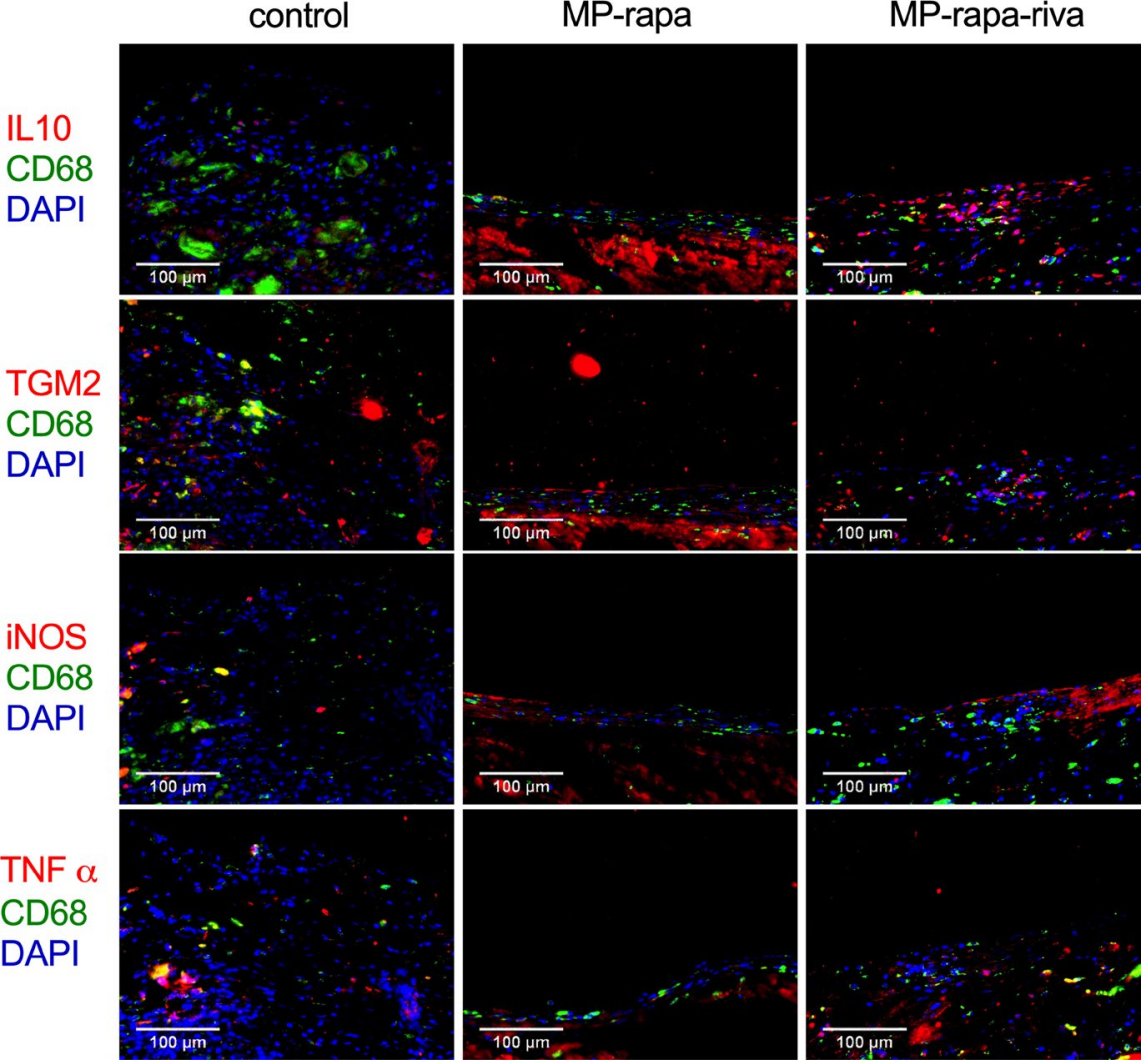
M1 and M2 macrophages in the neointima after rat IVC patch venoplasty at day 14. Photographs of the immunofluorescence of the neointima, first row, CD68(green), IL10 (red) and DAPI (blue); second row, CD68(green), TGM2(red) and DAPI (blue); third row, CD68 (green), iNOS (red) and DAPI (blue); forth row, CD68(green), TNFα (red) and DAPI (blue); scale bar, 100 μm; *n* = 3.

There were CD31 positive cells on the luminal side of the neointima, immunohistochemistry showed a similar reendothelialization rate in these three grafts (Figure 5A, 5B); there were also α-actin positive cells in the neointima (Figure 5A). Immunofluorescence showed phospho-mTOR (p-mTOR) expression in all three grafts, and there was a significantly smaller amount of PCNA positive cells in the neointima in the MP-rapa and MP-rapa-riva grafts (Figure 5C, 5D). M1 and M2 macrophages play a role in neointima hyperplasia, there were macrophages in the neointima in these three grafts, M2 type (CD68 and IL-10 dual positive, CD68 and TGM2 dual positive), M1 type (CD68 and iNOS dual positive, CD68 and TNF α dual positive) macrophages were interspersed in the neointima (Figure 6).

## Discussion

In this research, we showed that rivaroxaban and chitosan microparticle rapamycin coated polyglycolic acid (PGA) scaffold inhibits venous neointimal hyperplasia after patch angioplasty in a rat IVC venoplasty model. Although this is a small animal research, this broadens the application of rivaroxaban.

After vascular interventions, antiplatelet and anticoagulation drugs are used to prevent platelet accumulation and thrombus formation (Liu et al., 2019, Haybar et al., 2019). Neointimal cell, especially smooth muscle cells proliferation also plays an important role in the neointimal hyperplasia, so rapamycin or paclitaxel coated grafts are also widely used to inhibit neointimal cell proliferation (Bai et al., 2021). Recent studies showed programmed death-1 antibody or inhibitor coated vascular graft can also inhibit neointimal hyperplasia after patch angioplasty in rat (Sun et al., 2021, Bai et al., 2021). IL-33 antibody absorbed plant leaf patch can be used to inhibit venous neointimal hyperplasia in rat (Xie et al., 2021). But these interventions were all focused on one step in the neointimal hyperplasia; this may be the cause that the treatment of neointimal hyperplasia is not satisfactory in clinic. Although we developed a hierarchical hydrogel releasing system, but this system was complex. In this research, we fabricated this PGA scaffold patch which has dual antithrombosis and antiproliferation functions to decrease neointimal hyperplasia; this dual functional scaffold is much easier to make compared to the hierarchical hydrogel patch.

Heparin is still a widely used parenteral anticoagulant for prevention and treatment of thrombotic events, but heparin-induced thrombocytopenia (HIT) is a severe complication in clinic (Arepally and Ortel, 2006; Warkentin et al., 1998). Several clinical studies showed rivaroxaban may be an alternative option to heparin in patients with a history of HIT (Krauel et al., 2012; Sartori et al., 2015; Tardy-Poncet et al., 2015). Consistent with our research, other studies also showed that thrombosis and neointimal proliferation were significantly inhibited with antithrombotic agents such as FXa inhibitors (Iwatsuki et al., 2004) and P2Y12 inhibitors (Patil et al., 2010). These findings suggest that coagulation and platelet activation after vascular injury are critical for neointimal hyperplasia in animal models (Morishima and Honda, 2018).

Theoretically, hyaluronic acid rivaroxaban coating on the surface of the scaffold would release first after implantation to play a role of anticoagulation, we previously showed heparin coated decellularized human saphenous vein patch can decease neointimal thickness in a rat patch angioplasty model (Bai et al., 2020). Then the chitosan microparticle rapamycin would release from the interspaces of PGA scaffold to inhibit cell proliferation, this chitosan microparticle rapamycin has been widely investigated in basic researches (Chiesa et al., 2018, Yuan et al., 2008), we also used collagen as an adjuvant to secure chitosan microparticle rapamycin in the interspaces of the scaffold (Sareethammanuwat et al., 2020, Păun et al., 2020). Since we have previously showed rapamycin can inhibit venous neointimal hyperplasia in several researches, so we did not spend too much efforts on the function of rapamycin (Bai et al., 2017, Bai et al., 2021, Bai et al., 2021). We used rat IVC patch venoplasty model in this experiment because rivaroxaban is commonly used in the venous system, and the neointima is much thicker in the rat IVC venoplasty model compared to the aortic patch angioplasty model.

There were also limitations in this research: Firstly, the observation time is short (14 days) in this preliminary study, longer time of observation is needed to confirmed this dual functional vascular patch; secondly, the function of this coating technique need to be tested in large animal (pig or sheep) models; thirdly, more commonly used ePTFE and Dacron grafts should also be tested.

## Conclusion

We developed a new dual functional vascular patch to decrease thrombus formation and cell proliferation in a rat IVC venoplasty model. This is a preliminary small animal experiment, but this idea may have a potential clinical application.
